# BCMA CAR T cells in a patient with relapsing idiopathic inflammatory myositis after initial and repeat therapy with CD19 CAR T cells

**DOI:** 10.1038/s41591-025-03718-3

**Published:** 2025-04-17

**Authors:** Fabian Müller, Andreas Wirsching, Melanie Hagen, Simon Völkl, Carlo Tur, Maria Gabriella Raimondo, Jule Taubmann, Laura Bucci, Liang Zhang, Sascha Kretschmann, Michael Aigner, Markus Eckstein, Silvia Spörl, Soraya Kharboutli, Sebastian Böltz, Armin Atzinger, Luis Munoz, Georg Schett, Andreas Mackensen, Ricardo Grieshaber-Bouyer

**Affiliations:** 1 Department of Internal Medicine 5 – Hematology and Oncology, Friedrich-Alexander-Universität (FAU) Erlangen-Nürnberg and Universitätsklinikum Erlangen, Erlangen, Germany; 2 Deutsches Zentrum Immuntherapie (DZI), Friedrich-Alexander-Universität (FAU) Erlangen-Nürnberg and Universitätsklinikum Erlangen, Erlangen, Germany; 3 Department of Internal Medicine 3 – Rheumatology and Immunology, Friedrich-Alexander-Universität (FAU) Erlangen-Nürnberg and Universitätsklinikum Erlangen, Erlangen, Germany; 4 Department of Pathology, Friedrich-Alexander-Universität (FAU) Erlangen-Nürnberg and Universitätsklinikum Erlangen, Erlangen, Germany; 5 Department of Nuclear Medicine, Friedrich-Alexander-Universität (FAU) Erlangen-Nürnberg and Universitätsklinikum Erlangen, Erlangen, Germany

**Keywords:** Idiopathic inflammatory myopathies, Autoimmune diseases, Immunotherapy

## Abstract

CD19 chimeric antigen receptor (CD19 CAR) T cell therapy has been shown to induce stable drug-free remission in patients with refractory autoimmune disease. The management of potential relapses is currently unclear. Here we report on a 45-year-old woman with treatment-refractory Jo-1-associated anti-synthetase syndrome, who initially achieved disease remission after CD19 CAR T cell therapy but then experienced disease relapse after 9 months. After reinfusion of the same product, CAR T cells failed to expand and T cells targeting the CD19 CAR were detected. Despite full-dose lymphodepletion, no clinical response was observed. After bridging with anti-CD38 antibody daratumumab, which was efficacious with limited durability, plasma-cell-targeting B-cell maturation antigen (BCMA) CAR T cell therapy was performed. BCMA CAR T cells expanded, cleared plasma cells in lymphoid tissue, reduced autoantibody levels and re-induced stable drug-free remission. This case highlights the challenges in CAR T cell reinfusion, the potential of alternative targets and products, and suggests that the depletion of plasma cells may enhance therapeutic outcomes in patients who become treatment-refractory.

## Abstract (web summary)

In a patient with treatment-refractory idiopathic inflammatory myositis, reinfusion of CD19 CAR T cells following disease relapse led to the development of T cells targeting the CD19 CAR and no clinical improvement, with drug-free remission induced again after treatment with BCMA CAR T cells.

## Main

Chimeric antigen receptor (CAR) T cell therapy has revolutionized the treatment of hematologic malignancies, and has shown unprecedented effects in the treatment of drug-resistant severe autoimmune disease (AID)^1,2^. While long-standing drug-free remission of AID comes within reach with CAR T cell therapy, some patients may still experience disease relapse, which requires the development of new treatment concepts^3^. In this context, it is particularly interesting whether re-treatment with the original CAR T cell product or switching to a different product, potentially also switching the target, should be pursued instead.

**Fig. 1:**
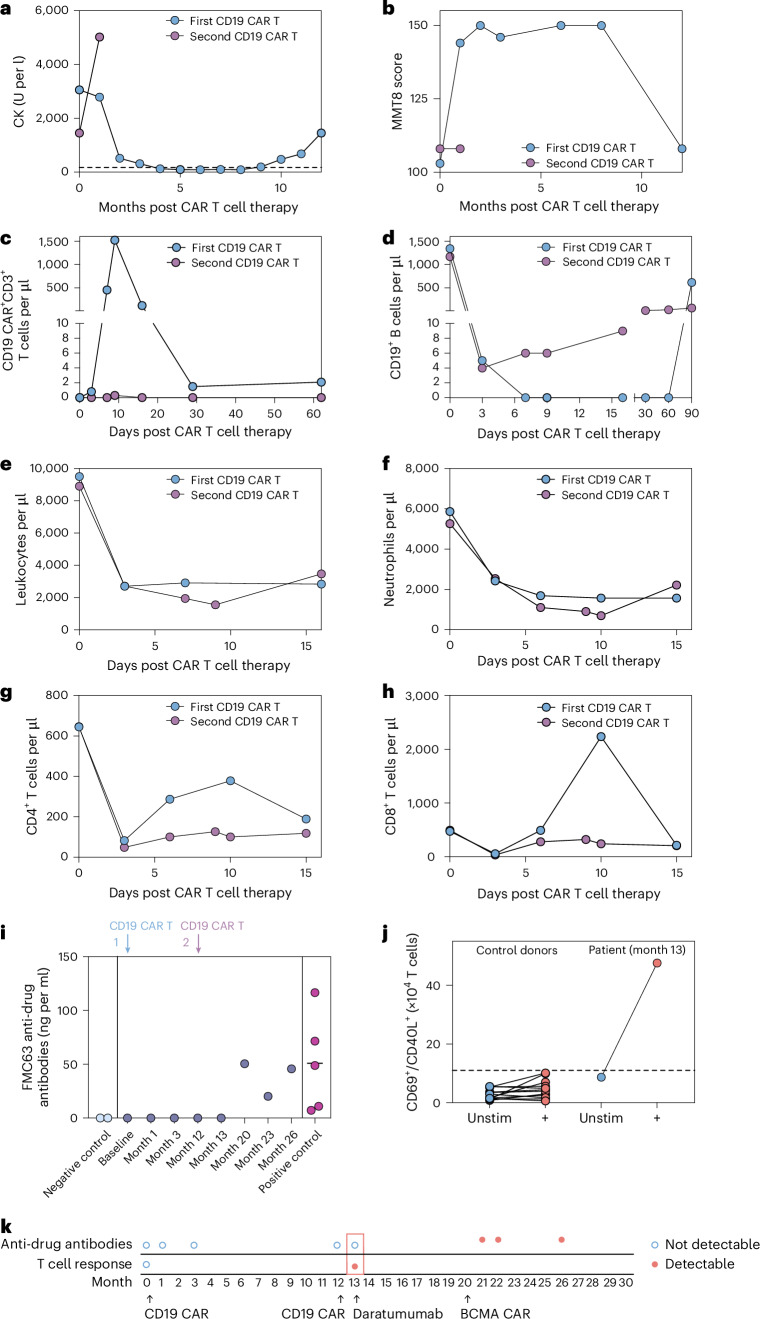
Relapse and repeated CD19 CAR T cell therapy in a patient with Jo-1^+^ IIM. **a**, Serum CK levels after CD19 CAR T cell therapy and at relapse. The horizontal dotted line indicates the upper limit of normal range (<170 U per l). **b**, Muscle strength as indicated by MMT8 score. **c**, CD19 CAR^+^CD3^+^ T cell counts in blood. **d**, CD19^+^ B cell counts in blood. **e**–**h**, Counts of leukocytes (**e**); neutrophils (**f**); CD4^+^ T cells (**g**) and CD8^+^ T cells (**h**) in blood after lymphodepletion (in cells per µl). **i**, ELISA quantification of the humoral response to CAR (FMC63). **j**, Quantification of the anti-CAR T cell response in control donors and the index patient. **k**, Sequence of events demonstrating the onset of the anti-CAR T cell response and humoral response. Unstim, no T cell stimulation; +, stimulated T cells.

**Extended Data Fig. 1:**
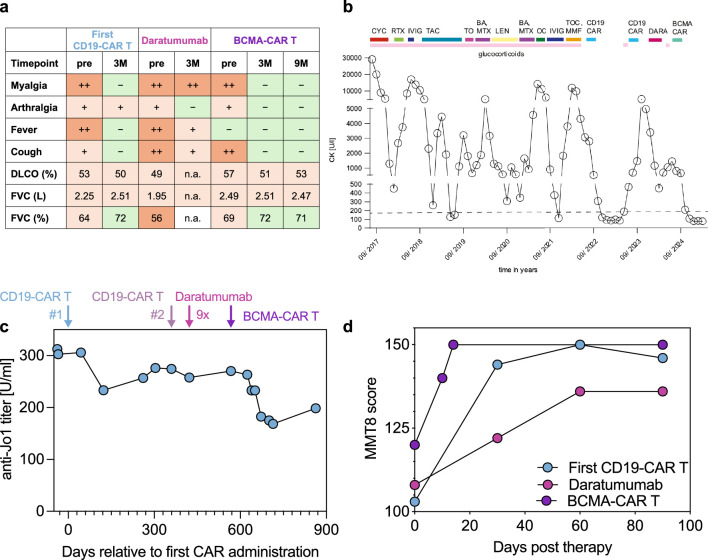
Clinical course of Jo-1+ anti-synthetase syndrome. **a**, Clinical manifestations before and three months after the first CD19-CAR T-cell therapy, daratumumab therapy and BCMA-CAR T-cell therapy. **b**, Serum creatinine kinase (CK) levels during various treatment regimens. The horizontal dotted line indicates the upper limit of normal range (<170 U/l). CYC = cyclophosphamide, RTX = rituximab, IVIG = intravenous immunoglobulins, TAC = tacrolimus, TO = tofacitinib, BA = baricitnib, MTX = methotrexate, LEN = lenabasum, OC = ocrelizumab, TOC = tocilizumab, MMF = mycophenolate mofetil, CAR = chimeric antigen receptor, DARA = Daratumumab. **c**, Longitudinal assessment of anti-Jo1 autoantibody titers. **d**, Muscle strength as indicated by Manual Muscle Testing 8 (MMT8) score.

Here we report a case of a 45-year-old woman with a 7-year history of idiopathic inflammatory myositis (IIM; Jo-1 positive anti-synthetase syndrome) who presented with myositis, arthritis, recurrent fever and severe progressive interstitial lung disease (ILD)^4^ (Extended Data Fig. 1). Previous treatment with intravenous immunoglobulins, tacrolimus, cyclophosphamide, mycophenolate, rituximab, ocrelizumab, tofacitinib, baricitinib and tocilizumab failed to induce disease remission (Extended Data Fig. 1). Due to ongoing activity, the patient received CD19 CAR T cell therapy (MB-CART19.1; 1.0 × 10^6^ viable CAR T cells per kg body weight). As previously reported (IIM patient 2 in ref. ^1^), CD19 CAR T cell therapy was efficacious, leading to a rapid normalization in circulating creatinine kinase (CK) levels (Fig. 1a) and muscle strength (Fig. 1b) and an improvement of ILD.

**Extended Data Fig. 2:**
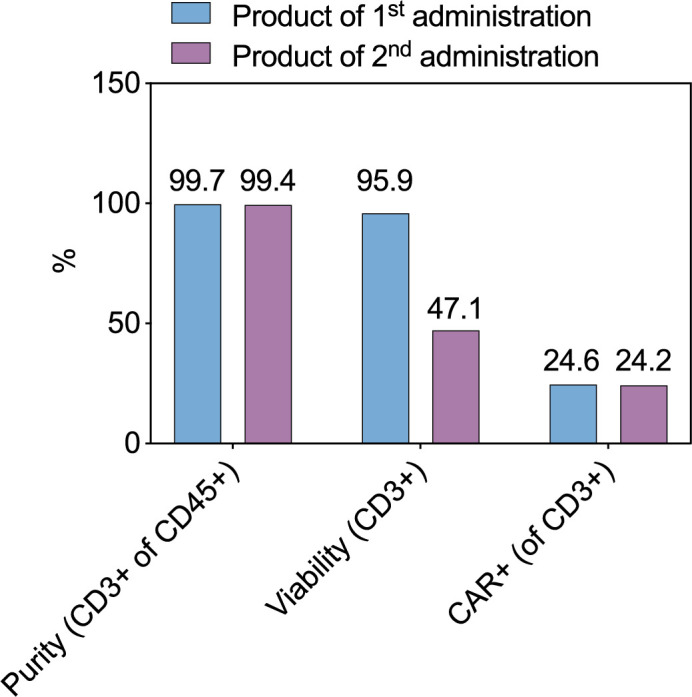
Quality of the CD19-CAR T-cell product at first and at second administration following cryopreservation. The purity (% CD3+ of CD45+), viability (% of CD3+) and CAR+ (% of CD3+) in the product was assessed by flow cytometry.

After a drug- and disease-free state of 9 months, the patient experienced gradual recurrence of myositis, with increasing myalgia, increasing CK level (Fig. 1a) and eventually worsening of muscle strength (Fig. 1b). No progression of ILD was observed. As musculoskeletal symptoms worsened and CK levels further increased despite glucocorticoid treatment, a second cycle of CD19 CAR T cell therapy was performed including full-dose lymphodepletion 12 months after the first application (1.3 × 10^6^ viable CAR T cells per kg body weight cryopreserved from the original stored product; Extended Data Fig. 2). Surprisingly, no CAR T cell expansion and no B cell depletion were observed, whereas the same CD19 CAR T cells had rapidly expanded during the initial administration (Fig. 1c) and had completely depleted circulating B cells (Fig. 1d). In contrast, virtually identical dynamics of leukopenia after lymphodepletion were observed (Fig. 1e–h). Despite similar leukocyte reduction, no therapeutic response was observed.

To investigate potential mechanisms of CAR T cell failure, we assessed anti-drug antibodies and specific T cell responses. No humoral response was detected in patient serum at the time of re-treatment (Fig. 1i). Cellular response was assessed using a proprietary peptide library containing 15-mers spanning the entire CAR sequence. This peptide library did not produce antigen-specific activation of patient T cells in the baseline sample but induced T cell activation in the follow-up sample, after failed expansion (Fig. 1j). Such reactivity towards 15-mers indicates the presence of anti-CAR-directed T cells, which have previously been described in rejection of CAR T cells after a second infusion and suggests acute rejection of CAR T cells before they could unfold their activity^5^. Of particular interest, the antigen booster via second CAR administration coupled with the incomplete depletion of B cells allowed the patient to subsequently develop a B cell response against the CAR (Fig. 1k).

**Extended Data Fig. 3:**
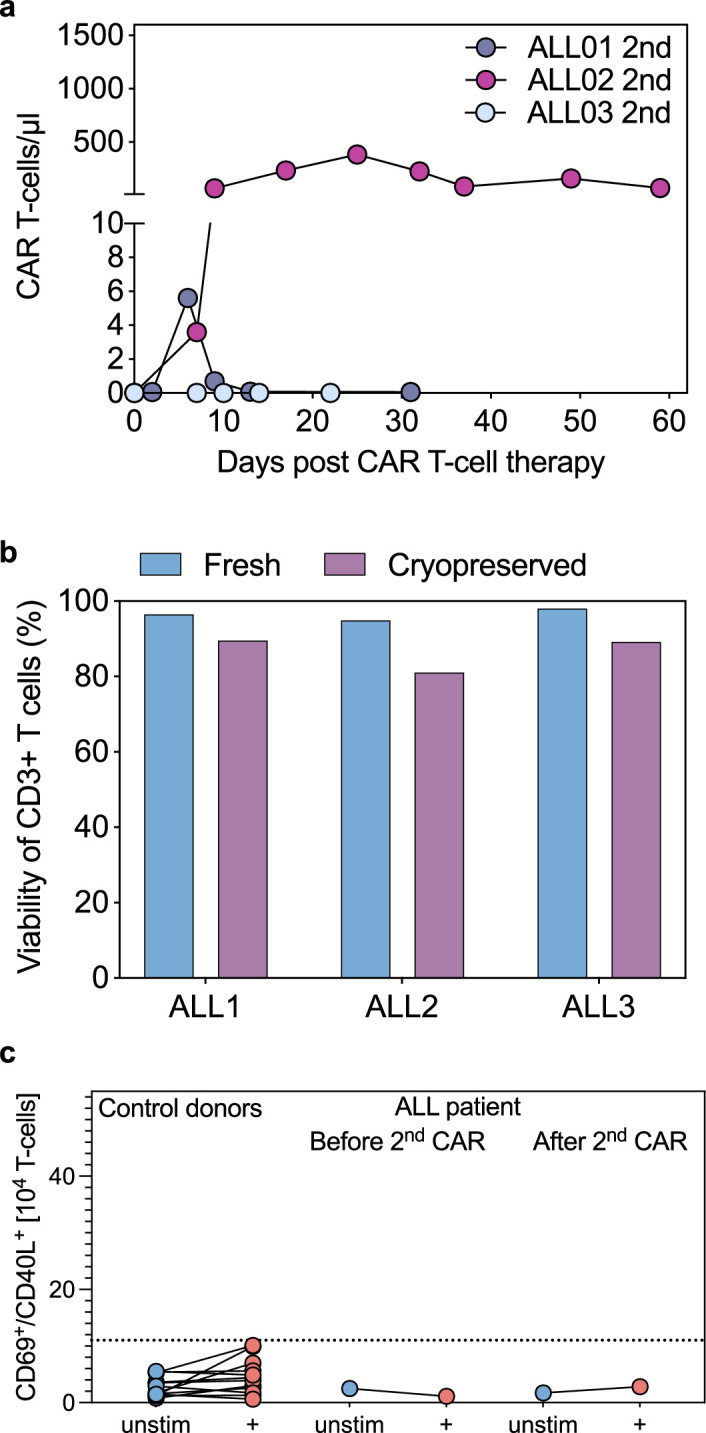
Re-treatment of patients with acute lymphoblastic leukemia (ALL) with cryopreserved CD19-CAR T-cells. **a**, Expansion dynamics of the second CD19-CAR T-cell therapy in three patients with acute lymphoblastic leukemia (ALL). **b**, Viability of CD3+ T-cells in the CD19-CAR T-cell product (live and after cryopreservation). **c**, Quantification of the anti-CAR T-cell response in N = 12 control donors and ALL patient 02.

In comparison, re-treatment of three patients with acute lymphoblastic leukemia (ALL) with a second dose of the same original CAR T cell product used in this IIM case (MB-CART19.1) led to CAR T cell expansion and clinical efficacy in two patients, while one patient showed no expansion shortly after B cell depletion with inotuzumab (Extended Data Fig. 3). These data from patients with ALL highlight the possibility of achieving therapeutic responses after a second exposure to the same CD19 CAR T cell product, which we did not observe in this patient with AID.

**Extended Data Fig. 4:**
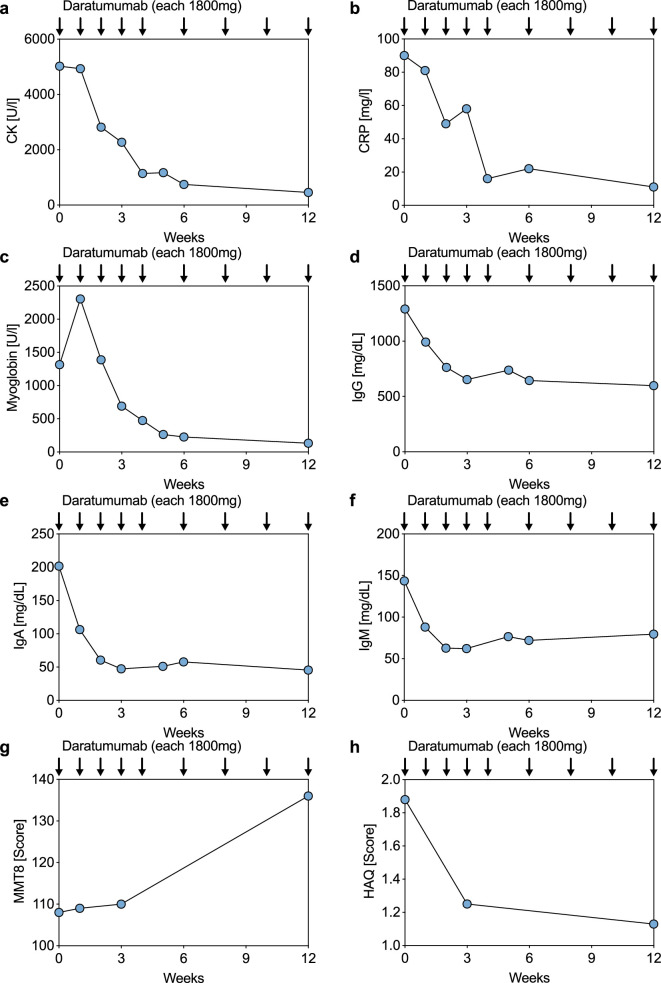
Serologic and clinical response in a patient with Jo-1 anti-synthetase syndrome to daratumumab therapy. A total of 9 doses of daratumumab were administered (1.8 g each: week 0, 1, 2, 3, 4, 6, 8, 10, 12) with dexamethasone premedication (20 mg initially, reduced subsequently to 8 mg). **a**, Serum creatinine kinase (CK) levels. **b**, C-reactive protein. **c**, Myoglobin level in the serum. **d**, Immunoglobulin G, **e**, Immunoglobulin A and, **f**, Immunoglobulin M levels in the serum. **g**, Muscle strength as indicated by Manual Muscle Testing 8 (MMT8) score. **h**, Health assessment score.

As re-treatment with CD19 CAR T cells had failed and disease remained continuously active, we next decided to target the plasma cell compartment with the anti-CD38 monoclonal antibody daratumumab. The decision to target plasma cells was also supported by the observation that anti-Jo-1 antibody responses did not decline in the patients after CD19 CAR T cell therapy, which spares plasma cells. Targeting CD38 with daratumumab affects plasmablasts and plasma cells next to B cells and has been used to treated patients with refractory forms of anti-synthetase syndrome and systemic lupus erythematosus (SLE)^6^. Nine bimonthly subcutaneous injections of daratumumab (1800 mg) together with dexamethasone (initially 20 mg, gradually tapered to 8 mg) were administered. During daratumumab therapy, the patient experienced cytomegalovirus-viremia, which was successfully treated with oral valganciclovir. Daratumumab therapy led to a decrease in CK, myoglobulin and C-reactive protein and improvement in muscle strength (Extended Data Fig. 4). However, myalgia and elevated CK values recurred after 5 months despite ongoing treatment.

**Fig. 2:**
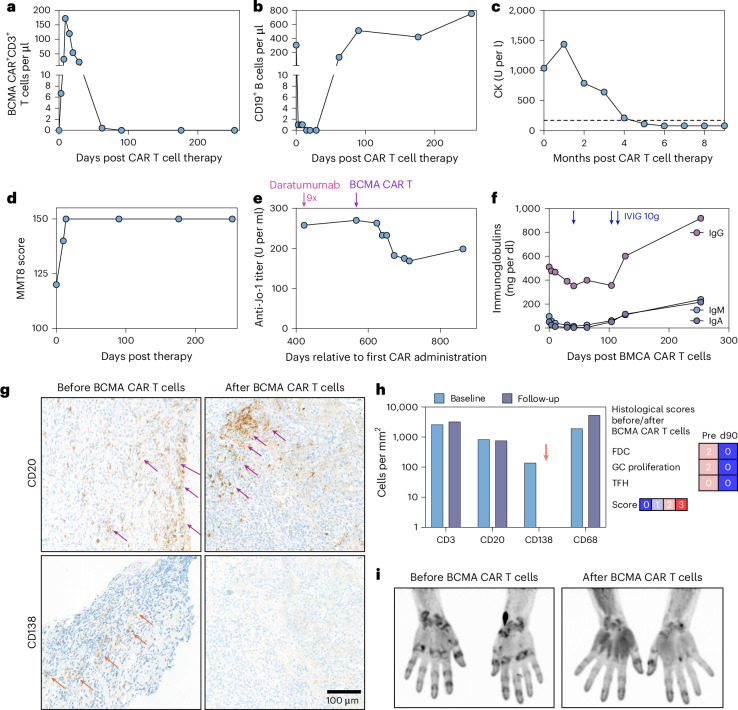
Treatment of anti-synthetase syndrome with BCMA CAR T cell therapy. **a**, BCMA CAR^+^CD3^+^ T cell counts in the blood. **b**, CD19^+^ B cell counts in the blood. **c**, Serum CK levels after CD19 CAR T cell therapy and at relapse. The horizontal dotted line indicates the upper limit of normal range (<170 U per l). **d**, Muscle strength as indicated by MMT8 score. **e**, Anti-Jo-1 autoantibody titer after BCMA CAR T cell therapy. **f**, Serum immunoglobulin levels after BCMA CAR T cell therapy. **g**, Representative staining of CD20^+^ B cells (purple arrows) and CD138^+^ plasma cells (orange arrows) in longitudinal lymph node biopsy before and after BCMA CAR T cell therapy. Images were acquired at ×400 magnification. Quantification was performed on the entire scanned tissue; one representative high-power field is shown. **h**, Quantification of CD3^+^ T cells, CD20^+^ B cells, CD138^+^ plasmablasts/plasma cells (cleared after BCMA CAR T cell therapy, orange arrow) and CD68^+^ macrophages along with the semiquantitative score^9^ for follicular dendritic cells (FDC), T follicular helper cells (TFH) and the germinal center (GC) proliferation index in longitudinal lymph node biopsy before and after BCMA CAR T cell therapy. **i**, Longitudinal FAPI-PET–CT before and after BCMA CAR T cell therapy. IVIG, intravenous immunoglobulins; Pre, before BCMA CAR T cell therapy; d90, 90 days after BCMA car T cell therapy.

**Extended Data Fig. 5:**
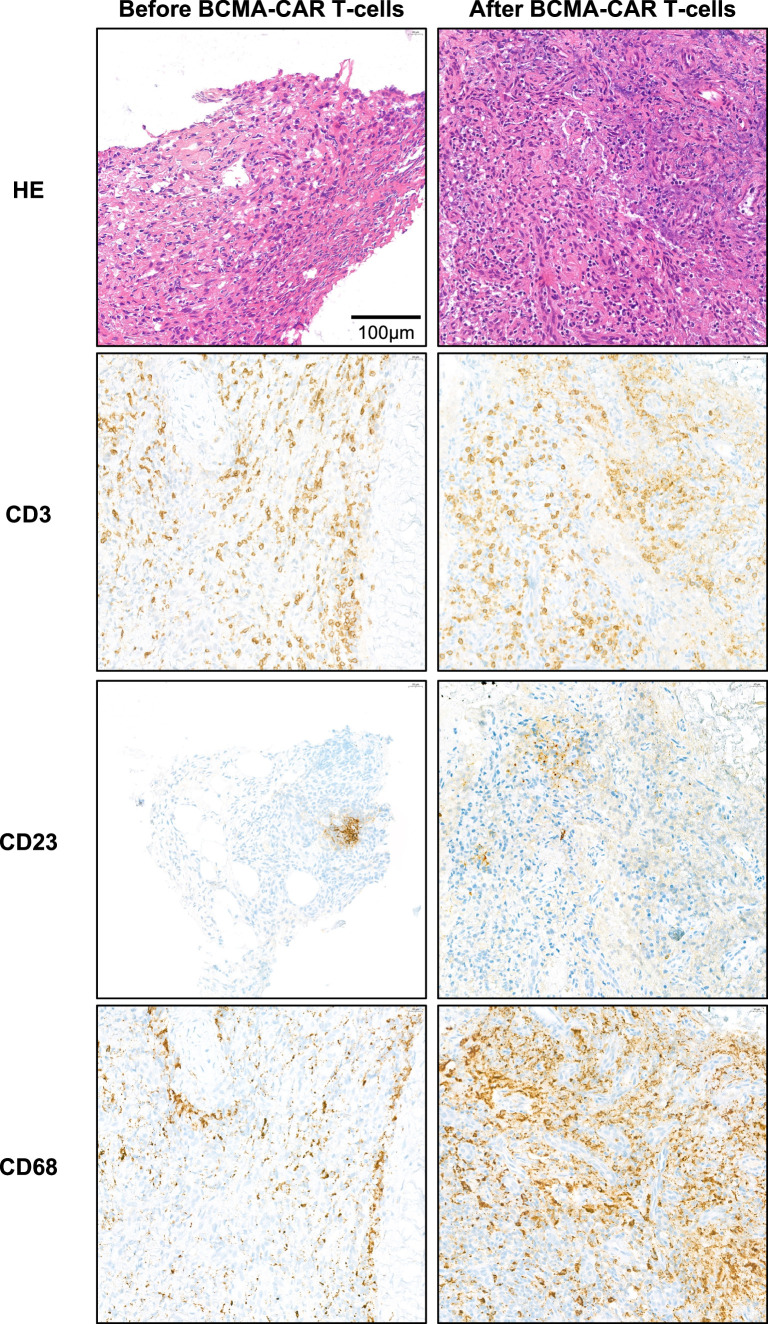
Representative histopathological staining of inguinal lymph node biopsy before and three months after BCMA-CAR T-cell therapy. Images were acquired at 400x magnification. Scale bar indicates 100 µm.

Due to ongoing disease activity and to maintain the plasma-cell-targeting treatment principle, we treated the patient with B-cell maturation antigen (BCMA) CAR T cells (idecabtagene vicleucel) under an expanded access program for critically ill patients in Germany. After lymphodepletion, 6 × 10^6^ viable BCMA CAR T cells per kg body weight were administered, 20 months after initial CAR T cell therapy. In contrast to the second CD19 CAR T cell product, BCMA CAR T cells expanded well and were detectable until day 62 (Fig. 2a). CD19^+^ B cells were depleted for 41 days (Fig. 2b), consistent with our previous observation that targeting BCMA in AID also depletes circulating B cells^7^. The patient experienced grade 1 cytokine release syndrome on day 3, which resolved upon a single dose of the anti-interleukin-6 receptor monoclonal antibody tocilizumab. No neuro- or hematotoxicity were observed and, supported by 100-day CMV-prophylaxis with letermovir, no infection occurred. CK normalized within 3 months (Fig. 2c) and major clinical response with disappearance of muscular impairment (Manual Muscle Testing 8 (MMT8) score: 150) was achieved after 3 weeks and maintained over 9 months (Fig. 2d). Notably, anti-Jo-1 autoantibodies declined after BCMA CAR T cell therapy, but did not seroconvert even after 9 months (Fig. 2e). While anti-ds-DNA antibodies are abrogated in patients with SLE treated with CD19 CAR T cells, other autoantibodies such as those against Jo-1 persist. It is therefore unlikely that the mere persistence of Jo-1 antibody is a marker of treatment resistance. However, longer follow-up is needed to confirm this. Concomitantly, the levels of IgG, IgA and IgM decreased (Fig. 2f) and IgG had to be substituted (10 g IgG after 6 weeks and 20 g IgG after 15 weeks). Inguinal lymph node biopsy revealed CD138^+^ plasma cells before BCMA CAR T cell therapy, which were completely cleared 3 months after therapy (Fig. 2g). In contrast, CD20^+^ B cells were not depleted in the lymph nodes (Fig. 2g,h and Extended Data Fig. 5). These data indicate that in the current patient BCMA-positive cells such as plasma cells may be involved in the disease process. That CD19-directed therapy was initially effective may be explained by the expression of CD19 by plasmablasts and some plasma cells. In line with clinically observed response, fibroblast activation protein inhibitor (FAPI)-positron emission tomography–computed tomography (PET–CT) showed remodeling in the muscle tissue but not in the lungs. FAPI signals related to arthritis were decreasing (Fig. 2i). The patient is now 9 months in drug-free remission and without any symptoms.

Together, although limited to a single patient, this case provides several new insights into IIM and CAR T cell therapy in AID in general. First, our data demonstrate that a switch of CAR T cell target can restore drug-free remission after relapse of AID after the first CAR T cell treatment; second, repeated treatment with the same CAR T cell product can be hampered by anti-CAR T cells preventing engraftment and third, immunosuppressive effects of lymphodepletion is not effective to influence AID in the absence of CAR T cell proliferation.

Following disease relapse after CAR T cell therapy, a different CAR T cell product restored stable drug-free remission. In contrast, the reuse of the original CAR T cell product may be complicated by an anti-CAR immune response, which can be mediated by T cells as observed here, or via antibodies. Both humoral and cellular immunity are also commonly observed in hematological use of CAR T cells^5^. In our case, it seems that T cells directed against the CAR had led to a failure of engraftment of infused CAR T cells, resulting in no expansion and no clinical response. We overcame this challenge by switching the CAR T cell product and the targeted antigen.

Whether lymphodepletion contributes to clinical response to CAR T cell therapy in AID has been a matter of debate. Lymphodepletion fosters homeostatic proliferation of CAR T cells and thereby contributes to their efficacy, but the direct immunosuppressive effect of lymphodepletion has also been considered to contribute at least to the short-term effects of CAR T cells. This case, with full-dose lymphodepletion but no proliferation of CAR T cell, provided a unique opportunity to dissect the effects. Notably, while full-dose lymphodepleting treatment decreased leukocytes, it had no clinical effect in the absence of CD19 CAR T cell expansion, suggesting that a single cycle of cyclophosphamide and fludarabine does not relevantly impact the course of AID.

Targeting the plasma cell compartment is a well-validated concept in the treatment of AID. Currently, most clinical data exist for the anti-CD38 monoclonal antibody daratumumab, which has been used in over 80 patients, including anti-synthetase syndrome^6^. Consistent with published results, daratumumab has been efficacious in our patient but the clinical effects were not sustained, potentially linked to insufficient depth of depletion required for long-term response. More recently, T cell engagers (TCEs) were used to induce clinical responses in patients with refractory autoimmune disease^7–9^. Compared to cell therapies, TCEs provide logistical advantages such as quick availability as an off-the-shelf drug, no necessity of lymphodepleting chemotherapy and more flexibility regarding dosing and re-treatment. The BCMA-targeting T cell engager teclistamab has shown promising efficacy in IIM, rheumatoid arthritis, systemic sclerosis, systemic lupus erythematosus and primary Sjogren’s syndrome, but long-term results are still pending and efficacy comparisons between TCEs and CAR T are therefore currently not possible.

BCMA CAR T cell therapy led to a robust and stable clinical response in our patient over 9 months. More patients and longer follow-ups are required to learn whether remission induced by BCMA CAR T cells is durable, that is longer than obtained after the first CD19 CAR T cell therapy. In support of a more durable remission, longitudinal histological analysis of inguinal lymph node biopsies revealed a complete depletion of CD138^+^ plasma cells after BCMA CAR T cell therapy, confirming effective targeting of plasma cells in lymphoid tissue. Interestingly, follicular dendritic cells also disappeared from the lymph node as it was shown after CD19 CAR T cell therapy^10^. Hence, deeper depletion of the plasma cell compartment might be beneficial for better disease responses in certain patients with AID. Notably, BCMA CAR T cells cleared only CD138^+^ but not CD20^+^ B cells from lymph nodes, while CD19 CAR T cells did not relevantly reduce CD138^+^ cells^10^.

The concept that a single treatment can induce long-lasting, drug-free remission is often described as ʽimmune resetʼ^11^. The clinical and molecular components of immune reset are not clearly defined. The term was originally coined in the context of autologous hematopoietic stem cell transplantation, which clears autoreactive lymphocytes including long-lived plasma cells, as noted by an elimination of protective vaccine titers, and leads to the subsequent formation of a new immune system from stem cells^12^. However, CD19 CAR T cell therapy also induces long-lasting drug-free remission after a short but deep clearance of B cells, followed by reconstitution with a naïve B cell compartment. In SLE, this is accompanied by seroconversion of dsDNA autoantibodies^1,2,13^. Other autoantibodies, such as Ro, La and Jo-1, appear to be more resistant to CD19 CAR T cell therapy, likely due to being anchored in long-lived plasma cells^14^. Concordantly, Jo-1 autoantibodies were remarkably resistant to depletion in this individual patient, while being completely abrogated in another patient with anti-synthetase syndrome treated with CD19 CAR T cells^1^. Notably, in this patient, a more pronounced decrease of Jo-1 autoantibodies was observed after the initial CD19 CAR T cell and particularly after BCMA CAR T cell treatment, compared to the unsuccessful second CD19 CAR T cell and daratumumab treatment. However, it is currently unclear whether seroconversion is required to reach long-standing clinical remission of AID, as the state of an immune reset may not solely depend on autoantibody status but also cellular effects such as antigen presentation and type I interferon responses. BCMA CAR T cell therapy decreased but did not abolish Jo-1 autoantibodies. These results mirror the behavior of vaccination-induced antibody titers, which typically are more reduced following BCMA-directed compared to CD19-directed CAR T cell therapy, but not fully abrogated^15^. Overall, sequential or simultaneous targeting of different B cell antigens might provide additional benefit in some AID patients^16^. However, specific safety aspects of BCMA CAR T cell therapy in autoimmune disease need to be considered, that is the loss of immunoglobulin production, which requires appropriate substitution and revaccination strategies to prevent severe infections.

## Methods

### Ethical approval for compassionate use

CAR T cell therapy was offered via a compassionate use program for critically ill patients according to the Arzneimittelgesetz, §21/2 and the Arzneimittel-Härtefall-Verordnung §2 that allows experimental treatment if (1) patients are afflicted by severe life-threatening disease, (2) have failed on previous treatments and (3) a scientific rationale exists for the potential efficacy of the respective treatment in the disease. Interventions are reported to the Legal Authorities (Paul Ehrlich Institute (PEI), Germany). Use of patient data and biomaterial is covered by License 334_18 B of the Institutional Review Board of the University Clinic of Erlangen. All procedures were performed in accordance with the Good Clinical Practice guidelines of the International Council for Harmonization and covered by license 334_18 B of the Institutional Review Board. All participants gave written informed consent according to CARE guidelines and in compliance with the Declaration of Helsinki principles. No commercial sponsor was involved. No participant compensation was offered.

### Inclusion and exclusion criteria

For inclusion, the patient had to have (1) a diagnosis of treatment-refractory idiopathic IIM, (2) a severe and progressive disease course and (3) resistance to multiple immunosuppressive treatments. Regarding severity and progression, the patient had to have histology proven and clinically active myositis and radiographic evidence of interstitial lung diseases (IIM). Regarding resistance, the patient had to have failed at least two immunosuppressive drug treatments. An Interdisciplinary Specialist Board consisting of rheumatologists and hematologist oncologists assessed and diagnosed the severity and resistance criteria of the screened patient.

Key exclusion criteria for patients were (1) anyone below the age of 18 years, (2) pregnant or lactating women, (3) anyone who did not understand the procedure and (4) anyone with more than one autoimmune disease or insufficiently defined autoimmune disease.

### CD19 CAR T cell therapy

CD19 CAR T cell therapy was performed as described^1^. Before leukapheresis, T cell targeted therapy was stopped for 3 weeks and prednisolone dose was reduced to less than 10 mg per day. Leukapheresis was performed at the collection site on day −14 according to local standard practice. The leukapheresis product was then transferred to the manufacturing site and used for the individual manufacturing of MB-CART19.1 using the automated CliniMACS Prodigy System. The manufacturing of MB-CART19.1 started on day −13 and finished on day −1.

The investigational medicinal product MB-CART19.1 consisted of autologous CD19 CAR transduced CD4/CD8 enriched T cells, derived from a leukapheresis product and processed using the CliniMACS Prodigy device (Miltenyi Biotec). Apheresis was done without prior cryopreservation within their shelf life by enrichment of CD4^+^ and CD8^+^CD3^+^ T cells. The cell count used in enrichment was limited to an upper level of 1.5 × 10^9^ cells. On day −12, cells were cultured in TexMACS media supplemented with IL-7 and IL-15 (Miltenyi Biotec) and human AB serum (ZKT). All materials used were fully good manufacturing practice compliant. For lentiviral transduction, a total cell count of 1 × 10^8^ cells was used as starting material. T cells were activated for transduction with polymeric nanomatrix conjugated to humanized CD3 and CD28 (T Cell TransAct; Miltenyi Biotec). Cells were transduced with a self-inactivating lentiviral vector expressing a CAR directed against human CD19. The second-generation lentiviral vector was kindly provided by Miltenyi Biotec. The vector encodes for a single-chain variable fragment, derived from the murine anti-human CD19 antibody FMC63, which binds to exon 4 of human CD19. Furthermore, it contains the information for a CD8^−^ derived hinge region, a TNFRSF19-derived transmembrane domain, a CD3ζ intracellular domain and a 4–1BB costimulatory domain. Cells were expanded for 12 days under clean room conditions at the certified good manufacturing practice laboratory of the Universitätsklinikum Erlangen (Department of Medicine 5, Hematology and Oncology) using the CliniMACS Prodigy system (Miltenyi Biotec) that performs all manufacturing steps in a single automated and functionally closed system. On day 5 of the manufacturing process the in-process control indicated if the manufacturing process was successful. Final release tests and in-process controls included cellular composition, transduction rate, viability, microbiological control, endotoxin and mycoplasma testing according to European Pharmacopoeia MB-CART19.1 was produced for each patient individually (personalized therapy).

Lymphodepleting chemotherapy was not started until the manufacturer confirmed the positive result of the in-process control. Lymphodepleting chemotherapy was performed with fludarabine 25 mg per m^2^ per day intravenously (i.v.) on days −5, −4, −3 and cyclophosphamide 1,000 mg per m^2^ per day i.v. on day −3 before CAR T cell transfer. On day 0, the investigational medicinal product MB-CART19.1, consisting of autologous CD19 CAR transduced T cells at a dose of 1 × 10^6^ viable CAR T cells per kg body weight, was applied. CAR T cells were administered as a short infusion after prophylactic application of antihistamines and acetaminophen. Oral prophylaxis with acyclovir and cotrimoxazol was performed for at least 3 months following CAR T cell therapy. Once CD4 T cells were stable above 200 per μl, prophylaxis was stopped. Signs of cytokine release syndrome and immune effector cell-associated neurotoxicity syndrome were monitored daily during the inpatient stay over a period of 10 days.

### BCMA CAR T cell therapy

Leukapheresis product was shipped to the manufacturer (Bristol Myers Squibb) and Idecabtagene vicleucel was produced from the cells. After lymphodepletion with cyclophosphamide (300 mg per m^2^ per day; days −5 to −3) and fludarabine (30 mg per m^2^ per day; days −5 to −3), BCMA CAR T cells were administered at a dose of 463 × 10^6^ cells (relative dose: 6 × 10^6^ viable cells per kg body weight).

### Monitoring of CAR T cells and leukocyte subsets

**Extended Data Fig. 6:**
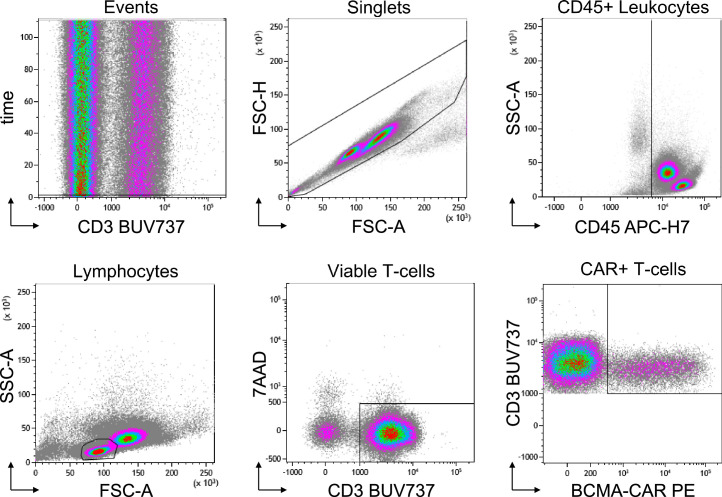
Flow cytometry gating strategy. Cells were gated as FSC-A/FSC-H single cells, CD45+ leukocytes, FSC/SSC lymphocytes, live (7AAD−) CD3+ T-cells and CAR+ cells.

Absolute cell counts were determined with BD Trucount tubes (BD Biosciences) according to manufacturer’s instructions. For monitoring of CAR T cells, peripheral blood mononuclear cells were isolated by density centrifugation, stained with CD19 CAR detection reagent, washed twice and stained with Biotin antibody, 7-AAD (BD Biosciences) and a standardized panel of antibodies against CD45, CD3, CD4 and CD8. The following anti-human antibodies were used for flow cytometry for monitoring leukocytes and CAR T cells after treatment: anti-CD3 (clone SK7), anti-CD4 (clone SK3), anti-CD8 (clone SK1), anti-CD19 (clone SJ25C1), anti-CD45 (clone 2D1) (all BD Biosciences), CD19 CAR Detection Reagent and Biotin antibody (clone REA746; both Miltenyi Biotec). Data were acquired on a LSR Fortessa (BD Biosciences) and analyzed by FlowJo v.10 software (Treestar). All measurements were taken from distinct samples. Flow cytometry measurements of leukocytes and CAR T cells were done at time of CAR T cell administration (day 0) and every 3 days for the first 9 days, thereafter weekly until month 1, monthly until month 3 and then every 3 to 6 months. Antibodies were used at 1:100 dilution. A representative gating strategy is shown in Extended Data Fig. 6.

### Immunohistochemistry and digital quantification of immune cells

Immunohistochemical stainings were performed using 2-µm-thick tissue sections transferred to positively charged adhesive slides (TOMO). All stainings were performed as prespecified assessments on a VENTANA BenchMark ULTRA autostainer platform (Ventana) according to accredited staining protocols in the routine immunohistochemistry facility of the Department of Pathology accredited and certified according to DIN EN ISO/IEC 17020. In brief, slides were cut, deparaffinized and further processed on the BenchMark ULTRA platform. Antigen retrieval was carried out with CC1 reagent (Ventana) with different incubation times and temperatures. Samples were stained for CD20 (surface antigen of B cells), CD68 (macrophages), CD138 (plasma cells) and CD23 (follicular dendritic cells). Stained slides were digitized using a Hamamatsu S210 slide scanner (Hamamatsu) at ×400 magnification and imported into the open source QuPath (v.0.4.3) digital slide analysis environment. The area of interest for cell quantification per square millimeter tissue area was defined as the synovial lining and the surrounding stroma, and annotated by a board-certified pathologist. CD19, CD20 and CD138 cell populations were quantified and validated by a pathologist and target cell counts per square millimeter region of interest area were reported.

### Measurement of FMC63 anti-drug antibodies using ELISA

Anti-FMC63 antibodies were detected using the ACROBIO Anti-CD19 (FMC63) CAR Immunogenicity ELISA Kit following kit instructions. Briefly, patient sera or positive control (provided anti-drug antibody) were coated overnight, FMC63 added, detected first with anti-FMC63 Biotin and stained with streptavidin-HRP. Color change of substrate was detected using an ELISA plate reader.

### Measurement of T cell mediated anti-CAR response

Cryopreserved peripheral blood mononuclear cells were collected from the patient 2 weeks after the second CD19 CAR T cell infusion. Control cells were obtained from healthy donors or patients before CAR T cell therapy. Peripheral blood mononuclear cells were stimulated with peptide pools with overlapping 15-mers spanning the entire CAR sequence. T cell activation was assessed as previously described^17^. Controls were used to identify a threshold for a positive response using mean + 3 s.d.

### Laboratory measurements

Levels of serum CK, CRP, myoglobulin, IgG, IgA and IgM were measured in routine laboratory. ELISA was performed to quantify Jo-1 autoantibodies (Orgentec).

### PET–CT examination of 68Ga-FAPI-04

Acquisitions of 68Ga-FAPI-04 were carried out on the same dedicated PET–CT system (Biograph Vision 600, Siemens Healthineers). The covered PET field-of-view was from skull to toes with an additional bed position of the hands (3 min per bed, axial field-of-view per bed 26.3 cm). PET data were corrected for random and scattered coincidences, as well as for decay during scanning. PET attenuation correction was carried out by the CT portion of the multimodal acquisition. All corrections and reconstructions were obtained using the PET–CT manufacturer’s software. PET–CT datasets were analyzed with commercially available software (syngo.via, Siemens Molecular Imaging), allowing review of PET, CT and fused imaging data. Visual evaluation was performed by two experienced nuclear medicine physicians and one radiologist. Datasets were analyzed by visual interpretation of coronal, sagittal and transverse slices.

### Statistical analysis

Data are derived from one patient with myositis and were not replicated. Sample sizes are as follows: Fig. 1a–h: *n* = 1 patient; Fig. 1i: *n* = 1 patient with myositis and *n* = 5 repeated measurements from one positive control patient (male, age 60 years); Fig. 1j: *n* = 1 index patient and *n* = 12 control donors (7 male, 5 female, age (median, range): 27.5, 21–47); Fig. 2: *n* = 1 patient; Extended Data Fig. 1: *n* = 1 patient; Extended Data Fig. 2: *n* = 1 patient; Extended Data Fig. 3a,b: *n* = 3 patients with ALL (male, 30 years; female, 32 years; male, 31 years); Extended Data Fig. 3c: *n* = 12 control donors (7 male, 5 female, age (median, range): 27.5, 21–47) as shown in Fig. 1j, one patient with ALL (female, 32 years); Extended Data Fig. 4: *n* = 1 patient; Extended Data Fig. 5: *n* = 1 patient; Extended Data Fig. 6: Representative gating strategy of *n* = 1 patient.

Data were analyzed with FlowJo v.10.6.1, GraphPad Prism v.10.4.1, QuPath v.0.4.3 and Kaluza v.2.1.

### Reporting summary

Further information on research design is available in the Nature Portfolio Reporting Summary linked to this article.

## Online content

Any methods, additional references, Nature Portfolio reporting summaries, source data, extended data, supplementary information, acknowledgements, peer review information; details of author contributions and competing interests; and statements of data and code availability are available at 10.1038/s41591-025-03718-3.

## Supplementary information

Reporting Summary

## Data Availability

Raw medical data is protected under the Patient Data Protection Act (PDSG). Data requests for patient-related laboratory measurements or clinical outcomes will be reviewed by the corresponding author within 4 weeks. Any data and materials that can be shared will be released via data transfer agreement.
