# Supplementary material for: BCMA CAR T cells in a patient with relapsing idiopathic inflammatory myositis after initial and repeat therapy with CD19 CAR T cells
Source: Nat Med. 2025 Apr 17;31(6):1793–7. doi: 10.1038/s41591-025-03718-3 (PMC12176613; doi:10.1038/s41591-025-03718-3)
Supplement: Supplementary file 1 — Reporting Summary [file 41591_2025_3718_MOESM1_ESM.pdf]

nature portfolio

Corresponding author(s): Ricardo Grieshaber-Bouyer

Last updated by author(s): Apr 15, 2025

Reporting Summary

Nature Portfolio wishes to improve the reproducibility of the work that we publish. This form provides structure for consistency and transparency in reporting. For further information on Nature Portfolio policies, see our Editorial Policies and the Editorial Policy Checklist.

Statistics

For all statistical analyses, confirm that the following items are present in the figure legend, table legend, main text, or Methods section.

n/a	Confirmed	
☐	☒	The exact sample size (*n*) for each experimental group/condition, given as a discrete number and unit of measurement
☒	☐	A statement on whether measurements were taken from distinct samples or whether the same sample was measured repeatedly
☒	☐	The statistical test(s) used AND whether they are one- or two-sided<br>*Only common tests should be described solely by name; describe more complex techniques in the Methods section.*
☒	☐	A description of all covariates tested
☒	☐	A description of any assumptions or corrections, such as tests of normality and adjustment for multiple comparisons
☐	☒	A full description of the statistical parameters including central tendency (e.g. means) or other basic estimates (e.g. regression coefficient) AND variation (e.g. standard deviation) or associated estimates of uncertainty (e.g. confidence intervals)
☒	☐	For null hypothesis testing, the test statistic (e.g. *F*, *t*, *r*) with confidence intervals, effect sizes, degrees of freedom and *P* value noted<br>*Give P values as exact values whenever suitable.*
☒	☐	For Bayesian analysis, information on the choice of priors and Markov chain Monte Carlo settings
☒	☐	For hierarchical and complex designs, identification of the appropriate level for tests and full reporting of outcomes
☒	☐	Estimates of effect sizes (e.g. Cohen's *d*, Pearson's *r*), indicating how they were calculated

Our web collection on statistics for biologists contains articles on many of the points above.

Software and code

Policy information about availability of computer code

Data collection	BD LSRFortessa, Hamatsu S210 slide scanner
Data analysis	FlowJo v10.6.1, GraphPad Prism v10.4.1, QuPath v0.4.3, Kaluza v2.1

For manuscripts utilizing custom algorithms or software that are central to the research but not yet described in published literature, software must be made available to editors and reviewers. We strongly encourage code deposition in a community repository (e.g. GitHub). See the Nature Portfolio guidelines for submitting code & software for further information.

Data

Policy information about availability of data

All manuscripts must include a data availability statement. This statement should provide the following information, where applicable:

- Accession codes, unique identifiers, or web links for publicly available datasets
- A description of any restrictions on data availability
- For clinical datasets or third party data, please ensure that the statement adheres to our policy

Raw medical data is protected under the Patient Data Protection Act (PDSG). Data requests for patient-related laboratory measurements or clinical outcomes will be reviewed by the corresponding author. Any data and materials that can be shared will be released via data transfer agreement.

Research involving human participants, their data, or biological material

Policy information about studies with human participants or human data. See also policy information about sex, gender (identity/presentation), and sexual orientation and race, ethnicity and racism.

Reporting on sex and gender	The patient described here is female. Control measurements shown in Figures 1i, 1j, 3a–c were taken from male and female donors/patients.
Reporting on race, ethnicity, or other socially relevant groupings	The patient described here is caucasian. No reporting on race from the control donors/patients is available.
Population characteristics	A 45-year old female, caucasian patient with Jo1 anti-synthetase syndrome received CD19-CAR T-cell therapy as part of a compassionate use program as described (Muller et al. NEJM 2024).
Recruitment	The patient presented to the Department of Medicine 3 – Rheumatology and Immunology at University Hospital Erlangen with refractory anti-synthetase syndrome. Due to severe, refractory disease, CAR T-cell therapy was initiated. No specific recruitment was performed, as this was not a clinical trial. A self-selection bias is unlikely but cannot be ruled out.
Ethics oversight	CAR T-cell therapy was offered via a compassionate use program for critically ill patients according to the Arzneimittelgesetz, §21/2 and the Arzneimittel-Härtefall-Verordnung §2 that allows experimental treatment if (i) patients are afflicted by severe life-threatening disease, (ii) have failed on previous treatments and (iii) a scientific rationale exists that potential efficacy of the respective treatment in the disease. Interventions are reported to the Legal Authorities (Paul Ehrlich Institute, PEI, Germany). Use of patient data and biomaterial is covered by license 334_18 B of the Institutional Review Board (IRB) of the University Clinic of Erlangen. All procedures were performed in accordance with the Good Clinical Practice guidelines of the International Council for Harmonization and covered by license 334_18 B of the Institutional Review Board (IRB). All participants gave written informed consent according to CARE guidelines and in compliance with the Declaration of Helsinki principles. No commercial sponsor was involved.

Note that full information on the approval of the study protocol must also be provided in the manuscript.

Field-specific reporting

Please select the one below that is the best fit for your research. If you are not sure, read the appropriate sections before making your selection.

☒ Life sciences ☐ Behavioural & social sciences ☐ Ecological, evolutionary & environmental sciences

For a reference copy of the document with all sections, see nature.com/documents/nr-reporting-summary-flat.pdf

Life sciences study design

All studies must disclose on these points even when the disclosure is negative.

Sample size	This is a case report of one patient, sample size was therefore limited to one and could not be influenced.
Data exclusions	Where available, repeat measurements are shown (Figures 1i–j, Extended Data Figure 3 a–c). No data was excluded.
Replication	There was no replication, as this is a case report of one patient.
Randomization	There was no randomization, as this was not possible in a single patient treated sequentially with multiple treatments, including cell therapy.
Blinding	There was no blinding, as this was not possible with a cell therapy product.

Reporting for specific materials, systems and methods

We require information from authors about some types of materials, experimental systems and methods used in many studies. Here, indicate whether each material, system or method listed is relevant to your study. If you are not sure if a list item applies to your research, read the appropriate section before selecting a response.

Materials & experimental systems

n/a	Involved in the study
☐	☒ Antibodies
☒	☐ Eukaryotic cell lines
☒	☐ Palaeontology and archaeology
☒	☐ Animals and other organisms
☐	☒ Clinical data
☒	☐ Dual use research of concern
☒	☐ Plants

Methods

n/a	Involved in the study
☒	☐ ChIP-seq
☐	☒ Flow cytometry
☒	☐ MRI-based neuroimaging

Antibodies

Antibodies used

Anti-CD3 (clone SK7), anti-CD4 (clone Sk3), anti-CD8 (clone sk1), anti-CD19 (clone SJ25C1), anti-CD45 (clone 2D1), CD19 CAR detection reagent and biotin antibody (clone REA746, Miltenyi Biotec, Bergisch-Gladbach, Germany).

Validation

All antibodies are validated for specificity to their respective target on human cells. The validation statements are available on the website of the manufacturer: https://www.miltenyibiotec.com/

Clinical data

Policy information about clinical studies

All manuscripts should comply with the ICMJE guidelines for publication of clinical research and a completed CONSORT checklist must be included with all submissions.

Clinical trial registration

N/A

Study protocol

N/A

Data collection

Data were collected from October 2022 to December 2024.

Outcomes

ACR/EULAR major response.

Plants

Seed stocks

Report on the source of all seed stocks or other plant material used. If applicable, state the seed stock centre and catalogue number. If plant specimens were collected from the field, describe the collection location, date and sampling procedures.

Novel plant genotypes

Describe the methods by which all novel plant genotypes were produced. This includes those generated by transgenic approaches, gene editing, chemical/radiation-based mutagenesis and hybridization. For transgenic lines, describe the transformation method, the number of independent lines analyzed and the generation upon which experiments were performed. For gene-edited lines, describe the editor used, the endogenous sequence targeted for editing, the targeting guide RNA sequence (if applicable) and how the editor was applied.

Authentication

Describe any authentication procedures for each seed stock used or novel genotype generated. Describe any experiments used to assess the effect of a mutation and, where applicable, how potential secondary effects (e.g. second site T-DNA insertions, mosiacism, off-target gene editing) were examined.

Flow Cytometry

Plots

Confirm that:

☒ The axis labels state the marker and fluorochrome used (e.g. CD4-FITC).

☒ The axis scales are clearly visible. Include numbers along axes only for bottom left plot of group (a 'group' is an analysis of identical markers).

☒ All plots are contour plots with outliers or pseudocolor plots.

☒ A numerical value for number of cells or percentage (with statistics) is provided.

Methodology

Sample preparation

Absolute cell counts were determined with BD TruCount tubes according to the manufacturer's instruction. For CAR T-cell monitoring, peripheral blood mononuclear cells (PBMC) were isolated from EDTA whole blood by gradient density centrifugation (Pancoll, PAN Biotech, Aidenbach, Germany) and directly analyzed or stored in liquid nitrogen until assayed. PBMC were washed in PBS and stained with the indicated antibodies according to the manufacturer's instructions.

Instrument	Samples were measured on a LSRFortessa (BD Biosciences, Heidelberg, Germany)
Software	Flow cytometry data were analyzed with FlowJo v10.6.1 and Kaluza v2.1.
Cell population abundance	1%–80%
Gating strategy	SSC-A <100,000; FSCA 50,000–150,000; FSC-H <100,000; CD45-APC-H7 > 1x10^4; CD3-BUV737 > 1x10^3; CAR-PE > 1 x 10^3

☒ Tick this box to confirm that a figure exemplifying the gating strategy is provided in the Supplementary Information.
